# Evaluating the Safety, Tolerability, and Disposition of Trazpiroben, a D_2_/D_3_ Receptor Antagonist: Phase I Single‐ and Multiple‐Ascending Dose Studies in Healthy Japanese Participants

**DOI:** 10.1002/cpdd.1057

**Published:** 2021-12-29

**Authors:** Takayoshi Yamaguchi, Kentarou Kudou, Hiroyuki Okamoto, Chunlin Chen, Roger Whiting, Hisakuni Sekino

**Affiliations:** ^1^ Takeda Pharmaceutical Company Ltd. Osaka Japan; ^2^ PRA Development Center KK Osaka Japan; ^3^ Current address: Alexion Pharma GK Tokyo Japan; ^4^ Takeda Development Center Americas, Inc. Cambridge Massachusetts USA; ^5^ Current address: Bayer Pharmaceuticals Whippany New Jersey USA; ^6^ Altos Therapeutics LLC Los Altos California USA; ^7^ Houeikai Medical Corporation Sekino Clinical Pharmacology Clinic Tokyo Japan

**Keywords:** gastroparesis, pharmacodynamics, pharmacokinetics, safety, trazpiroben

## Abstract

Trazpiroben (TAK‐906) is a peripherally selective dopamine D_2_/D_3_ receptor antagonist being developed to treat chronic gastroparesis. This phase I, randomized, double‐blind, placebo‐controlled, single‐ and multiple‐ascending dose, parallel‐group study evaluated the safety, tolerability, pharmacokinetics, and pharmacodynamics of trazpiroben in healthy Japanese men. Findings were compared with those from a prior US trial in healthy individuals. Overall, 24 participants were enrolled into 3 cohorts (each n = 8). Per cohort, 6 participants received trazpiroben (cohort 1, 50 mg; 2, 100 mg; 3, 10 mg) once on day 1 and twice daily on days 3 through 7, and two received placebo. Trazpiroben was well tolerated, with no clinically meaningful adverse events observed. Following single‐ and multiple‐dose administration, trazpiroben was rapidly absorbed and eliminated (mean elimination half‐life, 1.89‐6.45 hours; median time to maximum serum concentration [steady state], 1.00‐1.25 hours). Serum prolactin increased with trazpiroben treatment (mean maximum serum concentration 93.32 ng/mL [10 mg] vs. 10.83 ng/mL [placebo]), illustrating receptor target engagement. Results reflected those from healthy US participants, indicating a lack of differences between these ethnic populations in trazpiroben disposition and safety profile. Trazpiroben may represent a promising therapy for chronic gastroparesis across different populations, with further evaluation ongoing in a phase IIb study (NCT03544229).

Gastroparesis is a chronic gastric motility disorder characterized by delayed gastric emptying in the absence of any mechanical obstruction.^1^, ^2^, ^3^ Symptoms, which tend to be chronic with periods of exacerbation, typically include early satiety, postprandial fullness, nausea and vomiting, bloating, and upper abdominal pain.^1^ The majority of cases are idiopathic or secondary to surgery or diabetes,^4^ with diabetic gastroparesis considered a serious disease complication.^5^ Notably, gastroparesis significantly impacts patient quality of life, disrupting daily activities, and even reducing the annual income of those affected.^6^

Despite the associated disease burden, epidemiological data concerning gastroparesis are relatively limited, owing to the nonspecific nature of symptoms and difficulties in diagnosis. One study estimated the prevalence of clinically confirmed gastroparesis in the United States (2007 data) at 24.2 per 100 000 persons.^7^ There are no data for the prevalence of gastroparesis in Japan, possibly reflecting the absence of a standardized, accurate diagnostic procedure, and a poor awareness of the disease across Asia. In a recent survey of 490 physicians (including 133 physicians from Japan), half stated that they diagnosed <5 cases per year, with a quarter never diagnosing gastroparesis.^8^ However, considering that approximately one‐third of patients with diabetes are thought to also have diabetic gastroparesis^5^ and that the prevalence of diabetes in Japan was estimated to be 7.9% in 2010 and was predicted to rise over the coming decade,^9^ there is the possibility that gastroparesis is frequently underdiagnosed in Japan.

Most current treatment strategies aim to increase gastric motility through a combination of dietary modifications, pharmacological intervention, and/or gastric electrical stimulation,^1^, ^4^ with antiemetics and prokinetics (such as D_2_/D_3_ receptor antagonists and 5‐hydroxytryptamine 4 receptor agonists) frequently being employed.^10^, ^11^, ^12^ In particular, D_2_/D_3_ receptor antagonists have been shown to reduce the symptoms of gastroparesis by establishing normal gastric myoelectric activity and resolution of gastric dysrhythmias, which may be more pertinent to reducing symptoms than increasing gastric emptying alone.^2^, ^10^, ^13^, ^14^ Nonetheless, current treatment options for gastroparesis are limited; 2 agents with dopamine receptor antagonist activity, metoclopramide and domperidone, are available for the symptomatic management of gastroparesis in the United States, but carry safety concerns, namely, the risk of serious central nervous system (CNS) and cardiac adverse events (AEs), respectively.^15^, ^16^, ^17^

Trazpiroben (previously referred to as TAK‐906 or ATC‐1906M) is a novel, peripherally selective dopamine D_2_/D_3_ receptor antagonist under development for the treatment of gastroparesis. In vitro studies have shown that trazpiroben is primarily metabolized through a non–cytochrome P450 (CYP) pathway (56.7%) by multiple cytosolic, nicotinamide adenine dinucleotide phosphate–dependent reductases, with the remaining metabolism occurring through CYP3A4 (25.8%), CYP2C8 (11.8%), and other CYPs (5.7%).^18^ Metabolite M23 is a ketone product of trazpiroben, with aldo‐keto reductases and short‐chain dehydrogenases/reductases primarily involved in M23 formation.^18^ In a study of trazpiroben 25 mg administered to healthy participants, metabolite to parent ratios were 10.6%, with levels remaining consistent following co‐administration of itraconazole.^19^ Investigation of drug‐drug interactions showed that trazpiroben does not result in any significant direct inhibition of several of the major CYP isoforms (CYP1A2, CYP2B6, CYP2C8, CYP2C9, CYP2C19, CYP2D6, and CYP3A), and does not induce CYP isoforms when administered at a clinically relevant dose (0.06 μM).^18^ Additionally, an examination of inhibition of the CYP elimination route using itraconazole resulted in no substantial change in plasma trazpiroben levels,^19^ suggesting a low risk of drug‐drug interaction via this mechanism. Trazpiroben has demonstrated selective antagonism of dopamine D_2_/D_3_ receptors in nonclinical in vivo studies, as well as being peripherally selective, and is thus unlikely to have CNS effects.^20^, ^21^ Trazpiroben was also shown to have low affinity for the human ether‐à‐go‐go–related potassium channel, with no indication of affecting QRS duration, corrected QT interval (QTc) duration, or electrocardiogram (ECG) measurements,^22^ suggesting it is unlikely to affect cardiac repolarization or be associated with cardiovascular toxicities. In the first‐in‐human, single‐ and multiple‐ascending dose phase I study conducted in 56 healthy participants in the United States (AT‐01C study; NCT03268941),^23^ single and multiple doses of trazpiroben were well tolerated in healthy, predominantly White or Black men and women, and no clinically meaningful adverse effects, such as cardiovascular effects, were observed across the whole dose range (5‐300 mg). Furthermore, a recent phase IIa study (NCT03268941) involving 51 patients with idiopathic or diabetic gastroparesis recruited from multiple US clinical research centers evaluated the safety, pharmacokinetic (PK), and pharmacodynamic (PD) profile of trazpiroben and did not identify any safety signals.^24^ Following prior analysis in US participants, further information is required on the safety, PK, and PD profile of trazpiroben in wider ethnic populations. The current study was conducted to evaluate the safety and tolerability, and the PK and PD of single and multiple oral doses of trazpiroben in healthy Japanese men. The study design was made consistent with that of the US AT‐01C study,^23^ to allow informal comparison of intrinsic similarities and differences between the 2 populations.

## Methods

### Study Design

This study was a phase I, randomized, double‐blind, placebo‐controlled, single‐ and multiple‐ascending dose, parallel‐group analysis conducted to assess the safety, tolerability, PK, and PD of trazpiroben administered as single and multiple doses in healthy male Japanese participants. A total of 24 participants were enrolled and split across 3 cohorts (each n = 8), who received trazpiroben in parallel. Participants in cohort 1 received trazpiroben 50 mg, while cohorts 2 and 3 received trazpiroben at a dose of 100 or 10 mg, respectively. In each cohort, 6 participants received active study drug and the other 2 received placebo (Table 1).

**Table 1:** Design of the Japanese Study

Trazpiroben Dose^a^, ^b^, ^c^		
Single‐dose period	Multiple‐dose period^d^	Subjects (n)
10‐mg single dose on day 1	10 mg twice daily for 5 days from days 3 through 7	6 trazpiroben 2 placebo
50‐mg single dose on day 1	50 mg twice daily for 5 days from days 3 through 7	6 trazpiroben 2 placebo
100‐mg single dose on day 1	100 mg twice daily for 5 days from days 3 through 7	6 trazpiroben 2 placebo

^a^ Dose escalation to 100‐mg dose was based on a full blinded review of safety and tolerability data until follow‐up assessments with 50‐mg dose. The 10‐mg dose cohort was conducted in parallel with the 50‐ and 100‐mg cohorts.

^b^ The study drug in the single‐dose period and all morning doses of the study drug in the multiple‐dose period were orally administered after a fast of at least 10 hours that continued for at least 4 hours after dosing, with water intake prohibited for at least 1 hour before and after dosing. The evening dose was administered 12 hours after the morning dose and at least 2 hours after the evening meal.

^c^ Dose level in cohort 2 could be changed within the range up to 100 mg, as appropriate, based on safety and PK data at the doses in preceding cohorts of this study, that is, 50 mg twice daily in cohort 1 or 10 mg twice daily in cohort 3 (if applicable).

^d^ An evening dose of study drug was not administered on day 7.

After a 27‐day screening period, participants received a single dose of blinded study drug on day 1 (single‐dose phase), followed by multiple doses of blinded study drug twice daily for 5 days from day 3 through day 7 (multiple‐dose phase), without an evening dose of the study drug on day 7. Follow‐up assessments occurred on day 14 (7 days after the completion of the last treatment dose). The study drug in the single‐dose phase and all morning doses of the study drug in the multiple‐dose phase were orally administered after a fast of at least 10 hours and that continued for at least 4 hours after dosing, with water intake prohibited for at least 1 hour before and after dosing. The evening dose was administered 12 hours after the morning dose and at least 2 hours after the evening meal. The design of the US AT‐01C study has been described previously,^23^ and a schematic is provided in Figure S1 for comparison.

The proposed starting dose was 50 mg twice daily and the maximum dose permitted was 100 mg twice daily. Administration of trazpiroben 10 mg in cohort 3 was conducted in parallel with the other 2 cohorts. The safety and PK data gathered at the trazpiroben 10‐mg and 50‐mg doses were used to inform dose escalation in cohort 2, where participants were able to receive any dose up to 100 mg. Dose escalation from 50 to 100 mg was permitted only following a fully blinded review of safety and tolerability data for the 10‐ and 50‐mg doses of trazpiroben. Any serious AE related to the study drug and any AE related to the study drug that made continued administration difficult were considered reasons not to escalate the dose.

The study was conducted in compliance with the institutional review board requirements stated in the Good Clinical Practice regulations and guidelines and was compliant with the principles expressed in the Declaration of Helsinki. The Houeikai institutional review board approved the clinical trial protocol, the investigator's brochure, a sample informed consent form, and other trial‐related documents, and the study was performed at the Houeikai Medical Corporation, Sekino Clinical Pharmacology Clinic. All patients provided written consent for inclusion in the study.

### Inclusion and Exclusion Criteria

The study included healthy Japanese men aged 20 to 60 years inclusive, with a body weight of 50 kg or higher and with a body mass index (BMI) of 18.5 to 25 kg/m^2^. Individuals with a history of seizure or tardive dyskinesia, hyperprolactinemia, pituitary adenoma, hypothyroidism, or any gastrointestinal disease that would be expected to influence the absorption of drugs were excluded, as were those with a family history of prolonged QT interval, a QTc using Fridericia's formula >450 milliseconds, previous gastric bypass surgery or current gastric band fitted, abnormal laboratory values of transaminase, bilirubin, or creatinine, or abnormal ECGs at screening or baseline before administration of the study drug. The inclusion and exclusion criteria used in the AT‐01C study have previously been described.^23^

### Safety and Tolerability

Safety measures evaluated in this study included AEs and physical and vital signs, in addition to ECG parameters. AEs were continuously monitored and assessed from the time the participant provided informed consent until follow‐up at day 14. Assessment of vital signs and ECG monitoring (standard 12‐lead ECG) were conducted before dosing and at 1, 2, 4, 6, and 24 hours after the single/morning dose on days 1, 2, 7, and 8. Vital signs were additionally assessed before the morning dose on days 3 through 6 and at follow‐up on day 14, while ECG monitoring was additionally assessed before dosing and at 1 and 2 hours after the morning dose on day 5.

### Pharmacokinetic Analysis

Blood samples for trazpiroben and its metabolite, M23 (pharmacologically inactive), were collected before dosing and at 0.5, 1, 1.5, 2, 3, 4, 6, 8, 12, 16, and 24 hours after the single/morning dose on days 1, 2, 7, and 8, as well as before the morning dose on days 3 through 6. Urine samples for PK analyses were collected before dosing and at 0 to 6, 6 to 12, and 12 to 24 hours after dosing on days 1 and 2, as well as at 0 to 6 and 6 to 12 hours after the morning dose on day 7.

Plasma trazpiroben and M23 were quantified using a validated high‐performance liquid chromatography with tandem mass spectrometric detection (LC‐MS/MS) methods. These entities, along with their internal standards ([^13^C]‐trazpiroben and [^13^C]‐trazpiroben‐M23), were isolated from human plasma using dipotassium ethylenediaminetetraacetic acid anticoagulant by protein precipitation. Extracted compounds were injected on reversed‐phase column and separated by a gradient mobile phase composed of acetonitrile, methanol, water, ammonium formate, and formic acid. Eluting compounds were detected and quantified by TurboIonSpray in the positive ion mode and multiple reaction monitoring at transitions 518.2→232.1 (trazpiroben) and 520.2→232.1 (trazpiroben‐M23). The calibration range was 0.05 to 50.00 ng/mL and the lower limit of quantification was 0.05 ng/mL for both analytes. Within‐day accuracy and precision were relative error (RE) –2.7% to 8.2% and precision <7.5% (trazpiroben); and RE –4.0% to 8.4% and precision <6.5% (trazpiroben‐M23). Between‐day accuracy and precision were RE 1.3% to 5.0% and precision <6.7% (trazpiroben); RE –0.7% to 2.4% and precision <7.0% (trazpiroben‐M23).

In urine, trazpiroben and its metabolite were quantified using a similar validated LC‐MS/MS method. Analytes and their internal standards were isolated from human urine by addition of acetonitrile. Extracted compounds were injected onto a reversed‐phase column and separated by a gradient mobile phase composed of acetonitrile, methanol, water, ammonium formate, and formic acid. Eluting compounds were detected and quantified by TurboIonSpray in the positive ion mode and multiple reaction monitoring at transitions 518.2→232.1 (trazpiroben), 520.2→232.1 (trazpiroben‐M23). The calibration range was 5.0 to 5000.0 ng/mL and the lower limit of quantification was 5.0 ng/mL, for both analytes. Within‐day accuracy and precision were RE –3.0% to 8.8% and precision <8.6% (trazpiroben); RE –6.4% to 6.7% and precision <5.7% (trazpiroben‐M23). Between‐day accuracy and precision were RE 0.0% to 3.4% and precision <6.2% (trazpiroben) and RE –3.0 to –0.5% and precision <4.7% (trazpiroben‐M23). LC‐MS/MS methods used for plasma and urine samples were validated by Covance Laboratories (Madison, Wisconsin).

Plasma PK parameters assessed included area under the concentration‐time curve (AUC), maximum serum concentration (C_max_), time to C_max_ (t_max_), and elimination half‐life (t_1/2z_), and urine measures such as total amount excreted, fraction of administered drug excreted in the urine and renal clearance. The PK parameters for trazpiroben and M23 were determined from the concentration‐time profiles for all evaluable participants using high‐performance liquid chromatography with LC‐MS/MS. Actual sampling times, rather than scheduled sampling times, were used in all computations involving sampling times.

### Pharmacodynamic Analysis

Serum prolactin concentrations were measured as a biomarker for dopamine D_2_ receptor antagonism. Samples were collected before dosing and at 1, 2, 4, 6, and 24 hours after the single/morning dose on days 1, 2, 7, and 8, immediately before the morning dose on days 3 through 6, and at follow‐up on day 14. PD parameters assessed included AUC and C_max_, and were calculated from serum prolactin concentrations.

As for the PK analyses, blood samples intended for prolactin concentration determination were processed to serum, frozen, and stored at –70°C prior to analysis. Serum samples were analyzed using the in vitro ADVIA Centaur prolactin assay (Erlangen, Germany). The assay range is 0.3 to 200 ng/mL, and assay precision is <10%. Further details on the ADVIA Centaur assay have previously been published as part of the US AT‐01‐C study.^23^

### Statistical Methods

The sample size of this study was considered sufficient for the evaluation of trazpiroben safety, tolerability, PK, and PD following oral single and multiple doses and was not based on statistical power considerations.

The PK and PD analysis sets consisted of participants who received at least 1 dose of the study drug, completed the minimum protocol‐specified procedures with no significant deviations, and who were evaluable for PK or PD analyses, respectively. The safety set consisted of all participants who received at least 1 dose of the study drug.

Continuous variables for ECG parameters, clinical laboratory evaluations and vital signs, and AEs were summarized by dose across both dosing phases for each scheduled sampling time using descriptive statistics. For categorical variables, shift tables summarized the number of participants in each category at each postbaseline visit by category at baseline for each dose, with data summarized using descriptive summary statistics.

Plasma PK parameters for trazpiroben and M23 were summarized by dose for each scheduled sampling time using descriptive statistics; geometric means and coefficients of variation were computed for C_max_ and AUC. Dose proportionalities for trazpiroben and M23 plasma exposures (C_max_ and AUC) were assessed statistically across dose levels using linear and power function models, while urine PK parameters for trazpiroben and M23 were summarized by dose using descriptive statistics. For PD analysis of serum prolactin, the observed values and their changes from baseline were summarized by dose for each scheduled sampling time using descriptive statistics.

## Results

### Participant Disposition and Baseline Demographics

In total, 24 participants were randomized; 18 received trazpiroben and 6 received placebo. Demographics were broadly comparable across dose groups (Table S1). Participants had a mean (standard deviation [SD]) age of 28.5 (5.8) years and a mean (SD) BMI of 21.8 (1.8) kg/m^2^. The Japanese participants in the present study were younger and had lower BMI than those in the US AT‐01C study.^23^

### Safety Analysis

There were no serious AEs or severe AEs in the study, and none of the participants discontinued treatment owing to AEs. Five treatment‐emergent AEs (TEAEs) were reported, all of which were mild in intensity, and none of which were reported in >1 participant (Table 2). Three participants receiving trazpiroben experienced TEAEs, all of which were reported during the multiple‐dose phase. There was 1 case of pharyngitis in a patient receiving trazpiroben 10 mg, and a single report in 1 participant each of increased creatinine and increased lactate dehydrogenase in the trazpiroben 100‐mg group, none of which were considered related to the study drug. There were no QTc prolongation–associated TEAEs, neurologic TEAEs, or hyperprolactinemia–associated TEAEs reported during the study. Furthermore, there were no clinically significant findings from laboratory tests, vital signs, or ECG monitoring in participants receiving trazpiroben vs those receiving placebo. Safety findings were comparable to the US phase I study, which also found oral administration of single or multiple doses of trazpiroben to be well tolerated.

**Table 2:** Overview of TEAEs in Japanese Participants

	Number of Participants (%)							
	Placebo (n = 6)		Trazpiroben 10 mg (n = 6)		Trazpiroben 50 mg (n = 6)		Trazpiroben 100 mg (n = 6)	
	Events	Participants	Events	Participants	Events	Participants	Events	Participants
Treatment‐emergent AEs	2	1 (16.7)	1	1 (16.7)	0	0 (0.0)	2	2 (33.3)
Related	2	1 (16.7)	0	0 (0.0)	0	0 (0.0)	0	0 (0.0)
Not related	0	0 (0.0)	1	1 (16.7)	0	0 (0.0)	2	2 (33.3)
Mild	2	1 (16.7)	1	1 (16.7)	0	0 (0.0)	2	2 (33.3)
Moderate	0	0 (0.0)	0	0 (0.0)	0	0 (0.0)	0	0 (0.0)
Severe	0	0 (0.0)	0	0 (0.0)	0	0 (0.0)	0	0 (0.0)
Leading to study drug discontinuation	0	0 (0.0)	0	0 (0.0)	0	0 (0.0)	0	0 (0.0)
Infections and infestations	0	0 (0.0)	1	1 (16.7)	0	0 (0.0)	0	0 (0.0)
Pharyngitis	0	0 (0.0)	1	1 (16.7)	0	0 (0.0)	0	0 (0.0)
Investigations	1	1 (16.7)	0	0 (0.0)	0	0 (0.0)	2	2 (33.3)
Alanine aminotransferase increased	1	1 (16.7)	0	0 (0.0)	0	0 (0.0)	0	0 (0.0)
Blood creatinine increased	0	0 (0.0)	0	0 (0.0)	0	0 (0.0)	1	1 (16.7)
Blood lactate dehydrogenase increased	0	0 (0.0)	0	0 (0.0)	0	0 (0.0)	1	1 (16.7)
Renal and urinary disorders	1	1 (16.7)	0	0 (0.0)	0	0 (0.0)	0	0 (0.0)
Proteinuria	1	1 (16.7)	0	0 (0.0)	0	0 (0.0)	0	0 (0.0)

AE, adverse event; TEAE, treatment‐emergent adverse event.

### Pharmacokinetic Analysis

#### Plasma Concentrations

**Figure 1:**
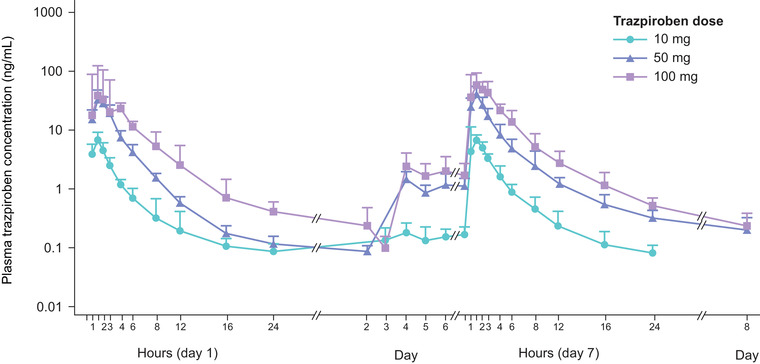
Mean (SD) plasma concentration–time curves of trazpiroben following single and multiple doses in Japanese participants. Plasma concentrations below the lower limit of quantification (0.05 ng/mL) were assigned a value of 0 ng/mL. SD, standard deviation.

**Figure 2:**
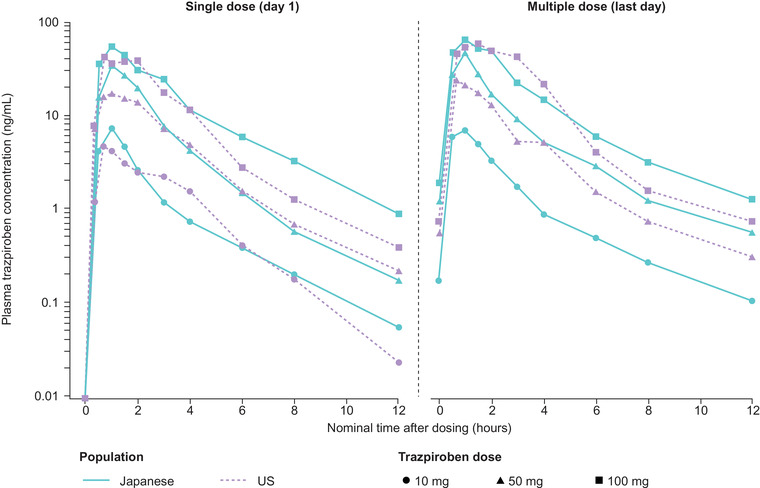
Mean plasma concentration–time curves of trazpiroben following single and multiple doses in Japanese and US participants. Predose samples were assigned a sampling time of 0 hours. Plasma concentrations below the lower limit of quantification (0.05 ng/mL) were assigned a value of 0 ng/mL.

Following single and multiple doses of trazpiroben, the plasma concentration of trazpiroben peaked ≈1 hour after administration and rapidly decreased thereafter on both day 1 and day 7, with a slightly higher mean plasma concentration of trazpiroben on day 7 than on day 1 (Figure 1). Similarly, the plasma concentration of metabolite M23 reached a peak ≈1.0 to 1.5 hours after administration and then swiftly fell on days 1 and 7, with a slightly higher mean plasma concentration on day 7 vs day 1 (Figure S2). For both trazpiroben and M23, geometric mean concentration slopes during the elimination phase were similar between day 1 and day 7 and between doses. Trazpiroben demonstrated rapid absorption and elimination over 24 hours in both Japanese and US populations (Figure 2).

#### Plasma PK Parameters

During the single‐ and multiple‐dose phases in Japanese participants, trazpiroben was shown to be rapidly absorbed (median t_max_, 1 hour; and median t_max_ at steady state, 1.0‐1.3 hours) and eliminated (mean t_1/2z_ 1.9‐5.2 hours [single‐dose phase] and 3.7‐6.5 hours [multiple‐dose phase] across dosing groups; Table 3). The observed mean C_max_ was 7.2 to 64.7 ng/mL during the single‐dose phase and C_max_ at steady state following multiple doses (C_max,ss_) was 8.0 to 81.8 ng/mL.

**Table 3:** Plasma PK Parameters of Trazpiroben in Japanese Participants

	Single‐Dose Phase (Day 1)					
	Trazpiroben 10 mg		Trazpiroben 50 mg		Trazpiroben 100 mg	
Variable	Japanese (n = 6)	US (n = 6)	Japanese (n = 6)	US (n = 6)	Japanese (n = 6)	US (n = 6)
C_max_, ng/mL						
Mean (SD)	7.2 (2.0)	6.2 (1.9)	36.9 (12.3)	22.0 (6.9)	64.7 (28.5)	48.3 (23.1)
%CV	28.4	30.2	33.2	31.3	44.0	47.8
t_max_, h						
Median	1.0	0.9	1.0	1.1	1.0	1.8
AUC_∞_, ng • h/mL						
Mean (SD)	13.9 (2.2)	13.1 (4.4)	74.1 (17.3)	56.7^a^ (11.6)	161.4 (38.1)	108.2^b^ (24.7)
%CV	15.6	33.8	23.4	20.4	23.6	22.9
AUC_last_, ng • h/mL						
Mean (SD)	13.6 (2.2)	12.9 (4.5)	73.4 (17.1)	53.2 (12.0)	159.6 (37.8)	103.8 (23.7)
%CV	16.0	34.7	23.2	22.6	23.7	22.8
AUC_24_, ng • h/mL						
Mean (SD)	13.8 (2.2)	13.1 (4.4)	73.6 (17.0)	53.2 (12.1)	159.6 (37.8)	103.8 (23.7)
%CV	15.9	33.8	23.0	22.7	23.7	22.8
t_1/2z_, h						
Mean (SD)	1.9 (0.9)	1.6 (0.2)	4.7 (3.8)	3.1 (0.4)^a^	5.2 (2.2)	5.4 (1.4)^b^
%CV	45.0	14.6	81.8	13.0	41.7	26.5
CL/F, L/h						
Mean (SD)	601.8 (92.2)	669.0 (166.8)	573.0 (110.7)	742.0^a^ (141.2)	539.0 (169.8)	791.0^b^ (196.9)
%CV	15.3	24.9	19.3	19.0	31.5	24.9

	Multiple‐Dose Phase (Last Day)					
	Trazpiroben 10 mg		Trazpiroben 50 mg		Trazpiroben 100 mg	
Variable	Japanese (n = 6)	US (NA)	Japanese (n = 6)	US (n = 6)	Japanese (n = 6)	US (n = 5)
C_max,ss_, ng/mL						
Mean (SD)	8.0 (2.9)	…	48.1 (12.2)	31.2 (9.6)	81.8 (20.0)	71.6 (20.9)
%CV	35.6	…	25.3	30.8	24.4	29.2
t_max,ss_, h						
Median	1.0	…	1.0	1.1	1.3	1.5
AUC_τ,ss_, ng • h/mL						
Mean (SD)	16.4 (1.7)	…	89.4 (9.1)	…	186.8 (9.6)	…
%CV	10.2	…	10.2	…	5.1	…
t_1/2z_, h						
Mean (SD)	3.7 (2.9)	…	6.5 (2.5)	11.0^a^ (1.2)	4.4 (1.1)	6.2 (2.8)^a^
%CV	78.5	…	38.0	10.9	25.1	44.0
CL/F, L/h						
Mean (SD)	504.2 (50.8)	…	460.7 (48.6)	650.0^a^ (93.1)	438.2 (23.0)	404.0^a^ (108.4)
%CV	10.1	…	10.6	14.3	5.3	26.8

AUC_∞_, area under the curve from time 0 to infinity; AUC_τ,ss_, area under the curve during a dosing interval, at steady state; AUC_24_, area under the curve over 24 h; AUC_last_, area under the curve from time 0 to time of last measurable concentration; CL/F, clearance; C_max_, maximum plasma drug concentration; C_max_,_ss_, maximum plasma drug concentration during a dosing interval, at steady state; CV, coefficient of variation; PK, pharmacokinetic; SD, standard deviation; t_1/2z_, elimination half‐life; t_max_, time to maximum concentration; t_max,ss_, time to maximum concentration at steady state.

^a^ n = 4.

^b^ n = 5.

Geometric mean C_max_ and AUC during a dosing interval (AUC_τ_) values were generally similar between Japanese and US participants (Table 3). Distribution of the individual exposures from the single‐ and multiple‐dose studies overlapped in both Japanese and US participants, although the median values were slightly higher in Japanese than US individuals (Figure S3).

Minimal accumulation of trazpiroben was observed following multiple doses, with the mean (SD) accumulation ratio calculated from AUC_τ_ at steady state (AUC_τ,ss_) and AUC_T_ ranging from 1.2 (0.1) to 1.3 (0.4), and the mean (SD) accumulation ratio calculated from C_max,ss_ and C_max_ ranging from 1.1 (0.2) to 1.6 (1.2). Similarly, limited accumulation of M23 was also noted following multiple doses of trazpiroben, with the associated mean (SD) accumulation ratio calculated from AUC_τ_ values ranging from 1.3 (0.4) to 1.8 (0.6) and mean (SD) accumulation ratio calculated from C_max_ values of 1.1 (0.5) to 2.0 (1.0).

When considering the US AT‐01‐C study, mean plasma trazpiroben t_1/2_ during the multiple‐dose phase was slightly greater than observed in Japanese participants, at ≈11.0 and 6.2 hours for the 50‐mg twice‐daily and 100‐mg twice‐daily doses, respectively (Table 3). Following 5 days of twice‐daily doses, little accumulation was observed with trazpiroben 50‐ and 100‐mg doses in the US population, with mean (SD) accumulation AUC_inf_ and C_max_ ratios of 1.1 (0.1) and 1.4 (0.1) for the 50‐mg cohort and 1.4 (0.2) and 10.6 (0.4) for the 100‐mg cohort.

#### Dose Proportionality

Following single doses of trazpiroben, C_max_ and AUC of trazpiroben appeared to increase in a dose‐proportional manner (Figure S3), as did the C_max_ and AUC of M23. With respect to the multiple‐dose phase, the C_max,ss_ of trazpiroben increased dose proportionally, while AUC_τ,ss_ did so in an approximately dose‐proportional manner. A similar trend was observed for the C_max,ss_ and AUC_τ,ss_ of M23 following multiple trazpiroben doses (Table S2).

Among the US population in the AT‐01‐C study, C_max_ and AUC parameters increased in a dose‐proportional manner for all doses during the single‐dose phase (Figure S3), and AUC from time 0 to 12 hours after dosing and AUC from time 0 to the last measurable concentration showed roughly dose‐proportional increases between the 2 trazpiroben doses (50 and 100 mg) studied in the multiple‐dose phase.

#### Urine PK Parameters

Urine PK parameters following single and multiple doses are presented in Table S3. The mean fraction of administered drug excreted in the urine and renal clearance for trazpiroben and M23 were similar between day 1 and day 7 and between doses.

### Pharmacodynamic Analysis

**Figure 3:**
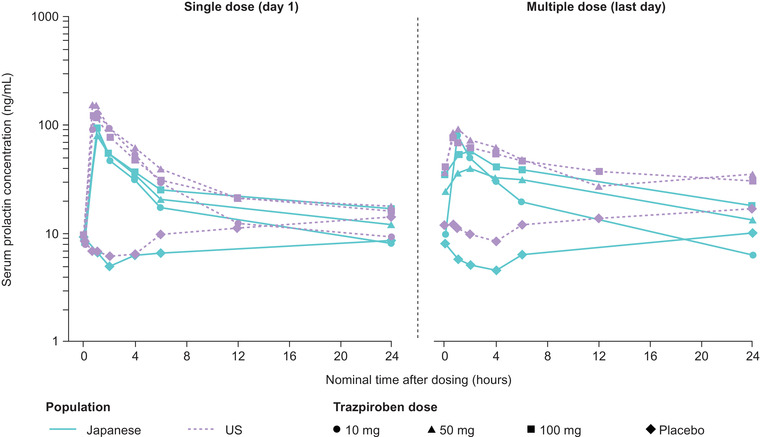
Mean serum concentration of prolactin following administration of single and multiple doses of trazpiroben in Japanese and US participants. Predose samples were assigned a sampling time of 0 hours.

Following administration of both single and multiple doses of trazpiroben, serum prolactin concentration exhibited a rapid increase immediately vs participants receiving placebo, followed by a slow decrease in concentration levels (Figure 3). Median t_max_ was 1.0 hour during the single‐dose phase and median t_max,ss_ was 1.0 to 2.0 hours following multiple dosing (Table 4). During the single‐ and multiple‐dose

**Table 4:** Serum Prolactin Parameters in Japanese Participants Following Administration of Trazpiroben

	Single‐Dose Phase (Day 1)							
	Placebo		Trazpiroben 10 mg		Trazpiroben 50 mg		Trazpiroben 100 mg	
Variable	Japanese (n = 6)	US (n = 14)	Japanese (n = 6)	US (n = 6)	Japanese (n = 6)	US (n = 6)	Japanese (n = 6)	US (n = 5)
C_max,_ ng/mL								
Mean (SD)	10.8 (2.0)	16.1	93.3 (45.4)	134.3 (85.0)	79.0 (37.4)	188.9 (172)	93.6 (55.6)	118.1 (66.4)
%CV	18.0	…	48.7	63.3	47.3	90.9	59.4	56.2
t_max,_ h								
Mean (SD)	9.7 (11.3)	16.7	1.0 (0.0)	1.2 (0.4)	1.0 (0.0)	0.9 (0.2)	1.0 (0.0)	0.9 (0.2)
%CV	…	…	…	37.7	…	25.8	…	25.8
AUC_∞_, ng • h/mL								
Mean (SD)	NC	…	660.2 (217)	…	803.2 (133)	…	1396.0 (370)	…
%CV	NC	…	32.9	…	16.6	…	26.5	…
AUC_last_, ng • h/mL								
Mean (SD)	176.5 (26.6)	259.6	476.7 (176)	664.8 (352)	554.8 (121)	1017.9 (806)	660.5 (171)	676.1 (268)
%CV	15.1	…	35.1	52.9	21.8	79.2	25.9	39.8
AUC_24_, ng • h/mL								
Mean (SD)	176.5 (26.6)	259.5	476.7 (168)	664.8 (352)	554.8 (121)	1017.3 (806)	660.5 (171)	675.1 (269)
%CV	15.1	…	35.1	52.9	21.8	79.2	25.9	39.8
t_1/2z_, h								
Mean (SD)	NC	…	14.6 (7.5)	…	13.9 (3.0)	…	29.1 (18.9)	…
%CV	NC	…	…	…	…	…	…	…

	Multiple‐Dose Phase (Last Day)							
	Placebo		Trazpiroben 10 mg		Trazpiroben 50 mg		Trazpiroben 100 mg	
Variable	Japanese (n = 6)	US (n = 4)	Japanese (n = 6)	US (NA)	Japanese (n = 6)	US (n = 6)	Japanese (n = 6)	US (n = 6)
C_max,ss_, ng/mL								
Mean (SD)	11.9 (3.5)	16.6 (5.2)	78.8 (38.0)	NA	41.9 (16.0)	98.1 (44.1)	59.0 (20.9)	78.8 (43.3)^a^
%CV	29.4	31.1	48.3	NA	38.2	45.0	35.4	55.0^a^
t_max,ss_, h								
Mean (SD)	45.3 (61.5)	7.7 (5.5)	1.0 (0.0)	NA	2.0 (1.1)	0.9 (0.2)	1.7 (0.5)	0.9 (0.2)^a^
%CV	…	71.2	…	NA	…	25.3	…	23.9^a^
AUC_τ,ss_, ng • h/mL								
Mean (SD)	75.9 (9.8)	…	343.2 (148)	NA	374.8 (143)	…	490.5 (122)	…
%CV	12.9	…	43.0	NA	38.1	…	24.9	…
t_1/2z_, h								
Mean (SD)	…	…	84.7 (11.4)	NA	75.8 (19.2)	…	71.3 (25.4)	35.7 (34.7)^b^
%CV	…	…	…	NA	…	…	…	97.2^b^

AUC_∞_, area under the concentration‐time curve from time 0 to infinity; AUC_τ,ss_, area under the concentration‐time curve during a dosing interval, at steady state; AUC_24_, area under the concentration‐time curve over 24 hours; AUC_last_, area under the concentration‐time curve from time 0 to time of last measurable concentration; CL/F, systemic clearance; C_max_, maximum plasma drug concentration; C_max_,_ss_ maximum plasma drug concentration during a dosing interval, at steady state; CV, coefficient of variation; NA, not applicable; NC, not calculated; SD, standard deviation; t_1/2z_, elimination half‐life; t_max_, time to maximum concentration; t_max,ss_, time to maximum concentration at steady state.

^a^ n = 5.

^b^ n = 2.

phases, a mean t_1/2z_ of 13.9 to 29.1 hours and 71.3–84.7 hours was observed, respectively. With trazpiroben doses of 50 and 100 mg, the peak mean serum prolactin concentration on day 7 was lower than on day 1. Following trazpiroben dosing, mean C_max_ was 79.0 to 93.6 ng/mL during the single‐dose phase (vs 10.8 ng/mL [placebo]), and C_max,ss_ was 41.9 to 78.8 ng/mL (vs 11.9 ng/mL [placebo]). Mean C_max_ and AUC parameters of prolactin slightly increased with increasing dose of trazpiroben, although increases were less than dose‐proportional, suggesting an almost maximal response at the 10‐mg dose (Table 4). Little, if any, accumulation in serum prolactin was noted with multiple doses of trazpiroben.

Similarly, serum prolactin concentration did not increase in a dose‐proportional manner in the US study, with near maximal response also observed at the 10‐mg dose (single‐dose phase), and minimal accumulation seen after 5 days of twice‐daily doses. Upon comparison, the distribution of the individual prolactin C_max_ and AUC_τ_ values from both the US and Japanese studies overlapped (Figure S4).

## Discussion

Trazpiroben is a novel, peripherally restricted and selective dopamine D_2_/D_3_ receptor antagonist developed for the chronic treatment of gastroparesis, and has demonstrated advantageous safety, PK, and PD profiles in a single‐ and multiple‐ascending dose phase I study conducted in US participants.^23^ To assess the disposition and safety profile of trazpiroben in an ethnically different population, the current study was conducted in healthy Japanese men. Trazpiroben was shown to have a favorable safety profile for single oral doses of 10, 50, and 100 mg, and for twice‐daily administration at the same dose levels, with no new safety signals vs the associated US study.^23^ Following administration, C_max_ increased in a dose‐proportional manner, and AUC parameters increased in a dose‐proportional or approximately dose‐proportional manner for both trazpiroben and metabolite M23, with minimal to no accumulation of either following multiple doses. As D_2_ receptor antagonism results in release of prolactin from the pituitary, serum prolactin concentration was used in this study as a biomarker for trazpiroben target engagement.^25^ Rapid increases in serum prolactin concentration were noted for all doses of trazpiroben, verifying an on‐target effect, although increases in exposure were less than dose‐proportional and showed that the elicited response was almost maximal at the lowest 10‐mg dose. Additionally, minimal accumulation in serum prolactin was noted with twice‐daily doses of trazpiroben across all doses.

Findings were similar between the current study in Japanese participants and the phase I study in US participants, with trazpiroben similarly well tolerated in both populations. Compared with the Japanese‐only population in the present study, participants in the US study were predominantly White (51.8%) or Black/African American (42.9%), with only 1 Asian patient being enrolled. The PK parameters of trazpiroben were generally comparable between studies, although average plasma trazpiroben C_max_ and AUC values were slightly higher in Japanese than in US participants. Importantly, distribution of individual exposures following single and multiple trazpiroben dose phases overlapped in both Japanese and US participants, indicating a lack of differences in PK between the 2 different populations. Comparable trazpiroben PD profiles were also observed for Japanese and US participants. In both studies, target engagement was evaluated using prolactin release, with near maximal response achieved at a 10‐mg dose of trazpiroben, and minimal accumulation of serum prolactin following multiple doses. The distribution of the individual prolactin C_max_ and AUC_τ_ values from the single‐ and multiple‐dose phases overlapped in both populations, indicating further similarities in the PD profile of trazpiroben between healthy Japanese and US participants.

In Japan, similarly to the rest of the world, underdiagnosis or challenges in diagnosis of gastroparesis in Japanese patients may stem, in part, from symptom overlap between gastroparesis and other conditions, such as functional dyspepsia, which are poorly recognized by Japanese physicians.^8^, ^26^, ^27^ A lack of approved, effective treatment for gastroparesis has also been cited as a reason why Japanese doctors were not interested in diagnosing gastroparesis^8^; for example, use of metoclopramide and domperidone is limited owing to safety concerns, namely, the risk of serious CNS and cardiac AEs, respectively.^28^, ^29^, ^30^ Prior preclinical and safety pharmacology analyses have shown minimal brain penetration of trazpiroben in rats and dogs, and no clinically meaningful impacts on CNS assessments in rats following dosing up to 1000 mg/kg/day.^21^, ^22^ Trazpiroben also has a weaker affinity for the human ether‐à‐go‐go–related channel than domperidone (half maximal inhibitory concentration = 15.6 μM vs 57 nM, respectively), and doses of trazpiroben between 5 and 300 mg were shown to have no clinically meaningful effect on QT interval in 72 healthy US participants in the associated US AT‐01C study.^23^ Following single and multiple doses of trazpiroben in the current analysis, no QTc prolongation–associated or neurologic TEAEs were observed, and no clinically significant differences in clinical laboratory tests, vital signs, or ECG monitoring were observed in participants receiving trazpiroben vs placebo. This is further supported by results from the US phase IIa study in which twice‐daily dosing of trazpiroben over 9 consecutive days was not associated with any clinically significant safety or tolerability issues.^24^ These findings suggest trazpiroben is a promising option for the chronic treatment of gastroparesis, and may offer an additional choice of therapy for patients and physicians in a currently limited field.

This study has several strengths. Administration of single and multiple doses of trazpiroben at 10, 50, and 100 mg allowed for comprehensive tolerability evaluation, and our findings indicate the therapy was well tolerated in a Japanese population. This study was designed so that the doses of trazpiroben and end points of interest were consistent with that of the US study, allowing informal comparison of the 2 populations. Comparison of these data with results from the US phase I study also serves to provide further context for our findings, and supports a favorable safety profile and disposition of trazpiroben across ethnically and racially diverse populations. One limitation of this study that must be acknowledged is that the population used for the current analysis was limited to healthy men. Sex‐related differences in the frequency of adverse drug reactions (ADRs) have been noted, with women more frequently experiencing ADRs than men.^31^, ^32^ PK differences between men and women represent a strong predictor of sex‐specific ADRs, underlining the importance of evaluating the PK and safety profiles of a therapy in both sexes.^31^ This is of additional significance for therapies such as trazpiroben that are intended to treat gastroparesis, as patients with the disease are predominantly women.^33^ To evaluate the safety and disposition of trazpiroben in wider populations, a phase IIa study has been completed in both male and female patients with gastroparesis (NCT03268941), with a phase IIb study currently ongoing (NCT03544229).

## Conclusion

Following administration of single and multiple doses to healthy Japanese men, the safety, PK, and PD profiles of trazpiroben were similar to those in healthy US participants, indicating a lack of differences between these ethnic populations. Accordingly, trazpiroben may offer a promising option for the chronic treatment of gastroparesis across different populations. Further studies are ongoing to evaluate trazpiroben in larger populations with gastroparesis.

## Conflicts of Interest

Y.T. and K.K. are employees of Takeda Pharmaceutical Company Ltd. and receive stock or stock options. O.H. was an employee of PRA Development Center KK at the time of the study. He is currently an employee of Alexion Pharma GK. C.C. was an employee of Takeda Development Center Americas, Inc., and received stock or stock options at the time of the study. He is currently an employee of Bayer Pharmaceuticals. R.W., a former shareholder of Altos Therapeutics LLC, will benefit from any future payments by Takeda with respect to certain clinical development and commercial milestones for trazpiroben. R.W. has received consulting fees from Takeda. S.H. is an employee of Houeikai Medical Corporation. Y.T., K.K., O.H., C.C., R.W., and S.H. have agreements with Takeda not to publish any Takeda‐sponsored research without prior authorization.

## Supporting information

Supporting information

Supporting information

Supporting information

Supporting information

Supporting information

Supporting information

Supporting information

Supporting information
