# Supplementary material for: Evaluating the Safety, Tolerability, and Disposition of Trazpiroben, a D2/D3 Receptor Antagonist: Phase I Single‐ and Multiple‐Ascending Dose Studies in Healthy Japanese Participants
Source: Clin Pharmacol Drug Dev. 2021 Dec 29;11(6):695–706. doi: 10.1002/cpdd.1057 (PMC9303893; doi:10.1002/cpdd.1057)
Supplement: Supplementary file 1 — Supporting information [file CPDD-11-695-s006.docx]

# Supplementary Figure 1. Study schematic for the Japanese (TAK-906-1004) and US (ATC-01C) studies.

PD, pharmacodynamic; PK, pharmacokinetic. ^a^ Single doses and all morning doses of trial medication in multiple-dose periods were preceded by a fast of at least 10 hours that continued for at least 4 hours after dosing.
^b^ Trazpiroben was administered twice daily 12 hours apart except on the last day (morning dose only).
^c^ In the Japanese study, single and multiple dosing was conducted sequentially for each participant.
^d^ In the US study, seven single-ascending dose cohorts (5 to 300 mg) were included (multiple-ascending dose cohorts were initiated after completion of all single-ascending dose cohorts); all available PK, PD, and safety data up to 48 hours post dose were reviewed before escalating the dose to that of the next sequential cohort. For this analysis, the 10 mg, 50 mg, and 100 mg cohorts were evaluated to enable comparison with the Japanese study dose cohorts.

# Supplementary Figure 2. Mean (SD) plasma concentration of M23 following single and multiple doses of trazpiroben in Japanese patients. SD, standard deviation.

# **Supplementary Figure 3**. Dose proportionality of C_max_ (a) and AUC_τ_ (b) for trazpiroben in Japanese and US participants. Trazpiroben 10 mg was not evaluated during the multiple-dose phase of the US study. Red circles indicate median value. AUC_τ_, area under the plasma concentration–time curve up to the last measurable concentration; C_max_, maximum plasma drug concentration.

# Supplementary Figure 4. C_max_ (a) and AUC_τ_ (b) of serum prolactin following single and multiple doses of trazpiroben in Japanese and US participants. Trazpiroben 10 mg was not evaluated during the multiple-dose phase of the US study. Red circles indicate median value. AUC_τ_, area under the plasma concentration–time curve up to the last measurable concentration; C_max_, maximum plasma drug concentration.
