# Supplementary material for: Evaluating the Safety, Tolerability, and Disposition of Trazpiroben, a D2/D3 Receptor Antagonist: Phase I Single‐ and Multiple‐Ascending Dose Studies in Healthy Japanese Participants
Source: Clin Pharmacol Drug Dev. 2021 Dec 29;11(6):695–706. doi: 10.1002/cpdd.1057 (PMC9303893; doi:10.1002/cpdd.1057)
Supplement: Supplementary file 2 — Supporting information [file CPDD-11-695-s005.pdf]

	Screening period	Randomization	Single-dose period		Multiple-dose period		Follow-up visit
			Dose[a]	Sample collection/safety assessment	Dose[a,b]	Sample collection/safety assessment/ discharge	
Japanese study[c]	Day −28 to −2	Day −1	Day 1	Day 2	Day 3 to 7	Day 8	Day 14
US study	Day −27 to −2	Day −1	Day 1	Day 1 to 2	Day 1 to 5	Day 1 to 5	Day 6 to 9

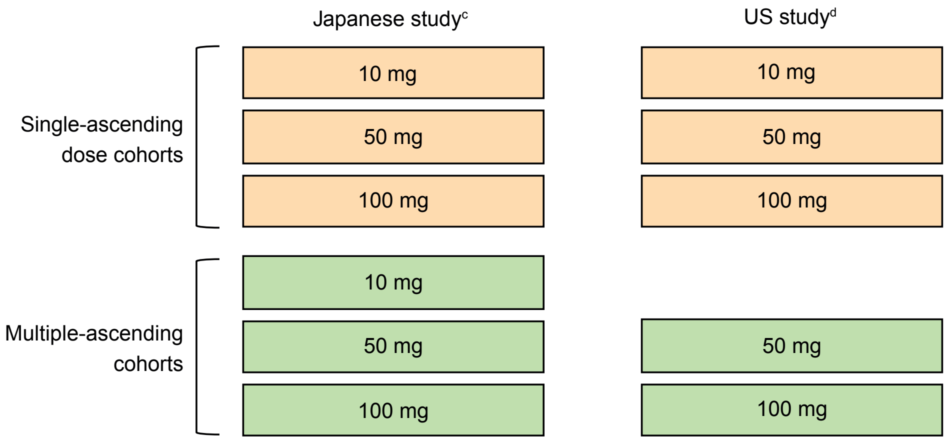

8 healthy participants per cohort: trazpiroben, n = 6; placebo, n = 2
(Total: 88 participants in 11 cohorts)
