# Supplementary figures and images for: Evaluating the Safety, Tolerability, and Disposition of Trazpiroben, a D2/D3 Receptor Antagonist: Phase I Single‐ and Multiple‐Ascending Dose Studies in Healthy Japanese Participants
Source: Clin Pharmacol Drug Dev. 2021 Dec 29;11(6):695–706. doi: 10.1002/cpdd.1057 (PMC9303893; doi:10.1002/cpdd.1057)

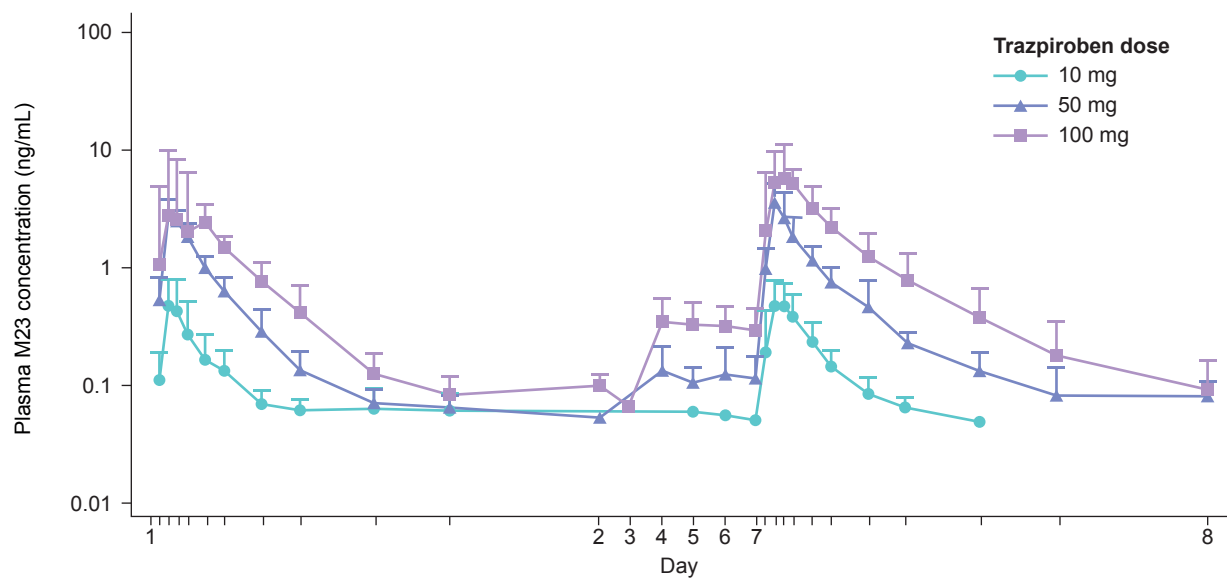

Supplement: Supplementary file 3 — Supporting information [file CPDD-11-695-s001.pdf]

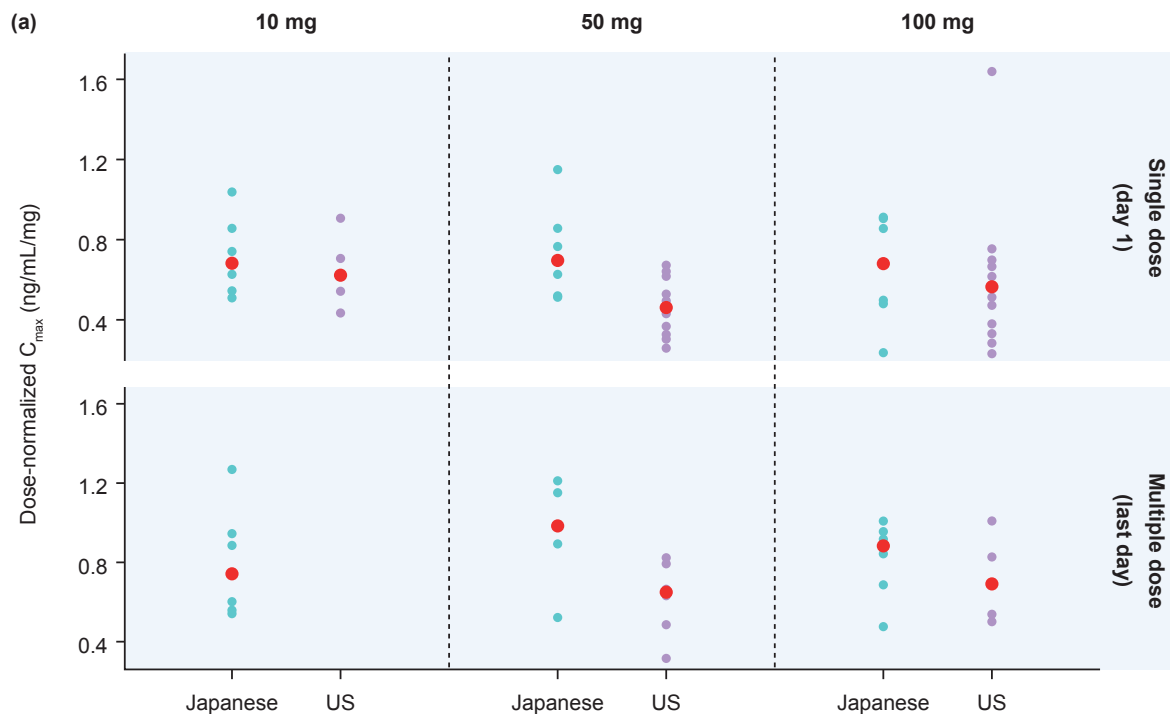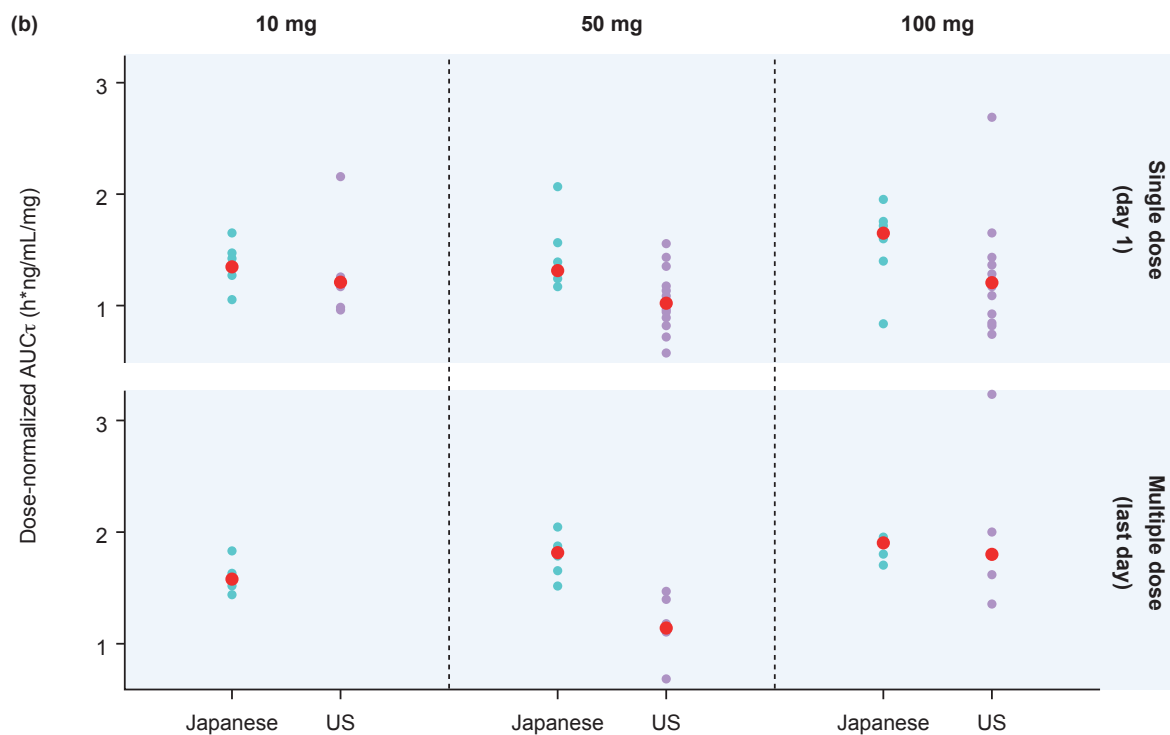

Supplement: Supplementary file 4 — Supporting information [file CPDD-11-695-s004.pdf]

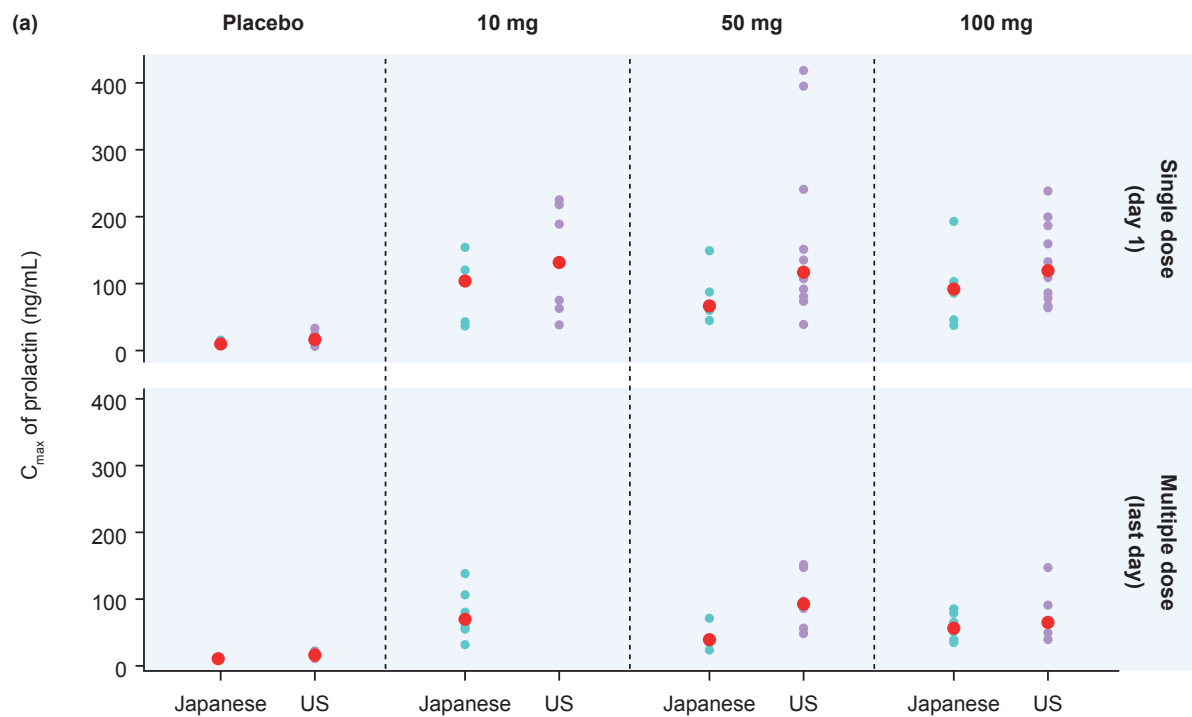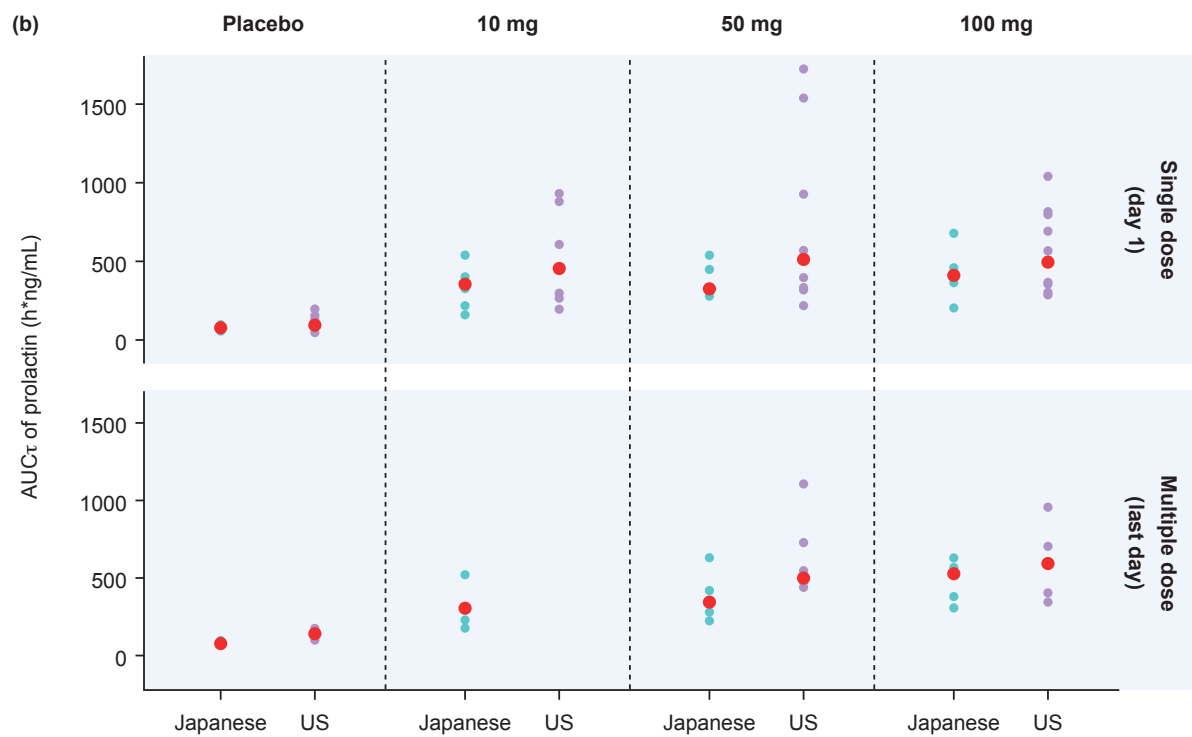

Supplement: Supplementary file 5 — Supporting information [file CPDD-11-695-s007.pdf]
