# Supplementary material for: Evaluating the Safety, Tolerability, and Disposition of Trazpiroben, a D2/D3 Receptor Antagonist: Phase I Single‐ and Multiple‐Ascending Dose Studies in Healthy Japanese Participants
Source: Clin Pharmacol Drug Dev. 2021 Dec 29;11(6):695–706. doi: 10.1002/cpdd.1057 (PMC9303893; doi:10.1002/cpdd.1057)
Supplement: Supplementary file 6 — Supporting information [file CPDD-11-695-s003.docx]

# Supplementary Table 1. Baseline Demographics in Japanese Participants.

|  | **Placebo**  **(n = 6)** | **Trazpiroben**  **10 mg**  **(n = 6)** | **Trazpiroben**  **50 mg**  **(n = 6)** | **Trazpiroben**  **100 mg**  **(n = 6)** | **Total**  **(N = 24)** |
| --- | --- | --- | --- | --- | --- |
| **Age, years** |  |  |  |  |  |
| Mean (SD) | 29.5 (6.8) | 29.0 (4.5) | 27.7 (4.7) | 27.8 (8.0) | 28.5 (5.8) |
| **Age, years, n (%)** | | | | | |
| Min≤ – <30 | 2 (33.3) | 3 (50.0) | 4 (66.7) | 4 (66.7) | 13 (54.2) |
| 30≤ – <40 | 4 (66.7) | 3 (50.0) | 2 (33.3) | 2 (33.3) | 11 (45.8) |
| 40≤ – ≤Max | 0 (0.0) | 0 (0.0) | 0 (0.0) | 0 (0.0) | 0 (0.0) |
| **Body weight, kg**  Mean (SD) | 63.2 (7.7) | 66.6 (5.4) | 63.1 (5.2) | 63.2 (6.9) | 64.0 (6.1) |
| **BMI, kg/m^2^** | | | | | |
| Mean (SD) | 21.8 (2.1) | 22.6 (1.7) | 21.4 (1.9) | 21.3 (1.8) | 21.8 (1.8) |
| **BMI, kg/m^2^, n (%)** | | | | | |
| Min≤ – <18.5 | 0 (0.0) | 0 (0.0) | 0 (0.0) | 0 (0.0) | 0 (0.0) |
| 18.5≤ – ≤25.0 | 6 (100.0) | 5 (83.3) | 6 (100.0) | 6 (100.0) | 23 (95.8) |
| 25.0< – ≤Max | 0 (0.0) | 1 (16.7) | 0 (0.0) | 0 (0.0) | 1 (4.2) |
| **Smoking classification, n (%)** | | | | | |
| Never | 6 (100.0) | 6 (100.0) | 6 (100.0) | 6 (100.0) | 24 (100.0) |
| Current | 0 (0.0) | 0 (0.0) | 0 (0.0) | 0 (0.0) | 0 (0.0) |
| Former | 0 (0.0) | 0 (0.0) | 0 (0.0) | 0 (0.0) | 0 (0.0) |
| **Consumption of alcohol, n (%)** | | | | | |
| Daily | 0 (0.0) | 0 (0.0) | 0 (0.0) | 0 (0.0) | 0 (0.0) |
| A few times per week | 0 (0.0) | 0 (0.0) | 0 (0.0) | 0 (0.0) | 0 (0.0) |
| A few times per month | 0 (0.0) | 0 (0.0) | 2 (33.3) | 2 (33.3) | 4 (16.7) |
| No | 6 (100.0) | 6 (100.0) | 4 (66.7) | 4 (66.7) | 20 (83.3) |
| **Consumption of caffeine, n (%)** | | | | | |
| Yes | 2 (33.3) | 2 (33.3) | 4 (66.7) | 2 (33.3) | 10 (41.7) |
| No | 4 (66.7) | 4 (66.7) | 2 (33.3) | 4 (66.7) | 14 (58.3) |

BMI, body mass index; Max, maximum; Min, minimum; SD, standard deviation.
