# Supplementary material for: Evaluating the Safety, Tolerability, and Disposition of Trazpiroben, a D2/D3 Receptor Antagonist: Phase I Single‐ and Multiple‐Ascending Dose Studies in Healthy Japanese Participants
Source: Clin Pharmacol Drug Dev. 2021 Dec 29;11(6):695–706. doi: 10.1002/cpdd.1057 (PMC9303893; doi:10.1002/cpdd.1057)
Supplement: Supplementary file 7 — Supporting information [file CPDD-11-695-s002.docx]

# Supplementary Table 2. Plasma PK Parameters of M23 in Japanese Participants

|  | **Single-dose phase (Day 1)** | | | | |
| --- | --- | --- | --- | --- | --- |
| **Variable** | **Trazpiroben**  **10 mg**  **(n = 6)** | | **Trazpiroben**  **50 mg**  **(n = 6)** | **Trazpiroben**  **100 mg**  **(n = 6)** | |
| **C_max_ (ng/mL)** |  | |  |  | |
| Mean (SD) | 0.6 (0.3) | | 3.1 (0.6) | 5.2 (3.0) | |
| %CV | 50.1 | | 20.2 | 58.6 | |
| **T_max_ (h)** |  | |  |  | |
| Mean (SD) | 1.2 (0.3) | | 1.3 (0.4) | 1.6 (1.2) | |
| %CV | – | | – | – | |
| **AUC_∞_ (h*ng/mL)** |  | |  |  | |
| Mean (SD) | 1.7 (0.5) | | 8.1 (2.1) | 17.3 (7.5) | |
| %CV | 28.1 | | 26.3 | 43.6 | |
| **AUC_last_ (h*ng/mL)** |  | |  |  | |
| Mean (SD) | 1.3 (0.6) | | 7.8 (2.1) | 16.7 (7.2) | |
| %CV | 45.9 | | 27.4 | 43.1 | |
| **AUC_24_ (h*ng/mL)** |  | |  |  | |
| Mean (SD) | 1.4 (0.6) | | 8.0 (2.1) | 16.9 (7.1) | |
| %CV | 42.4 | | 26.10 | 42.2 | |
| **t_1/2z_ (h)** |  | |  |  | |
| Mean (SD) | 3.2 (2.3) | | 2.5 (1.2) | 4.4 (3.1) | |
| %CV | – | | – | – | |
|  | **Multiple-dose phase (last day)** | | | | |
| **Variable** | **Trazpiroben**  **10 mg**  **(n = 6)** | **Trazpiroben**  **50 mg**  **(n = 6)** | | | **Trazpiroben**  **100 mg**  **(n = 6)** |
| **C_max,ss_ (ng/mL)** |  |  | | |  |
| Mean (SD) | 0.6 (0.3) | 4.4 (2.3) | | | 8.8 (4.1) |
| %CV | 42.1 | 51.7 | | | 46.4 |
| **T_max,ss_ (h)** |  |  | | |  |
| Mean (SD) | 1.2 (0.3) | 1.0 (0.3) | | | 1.4 (0.4) |
| %CV | – | – | | | – |
| **AUC_τ,ss_ (h*ng/mL)** |  |  | | |  |
| Mean (SD) | 1.7 (0.5) | 10.2 (2.8) | | | 26.6 (9.4) |
| %CV | 28.1 | 27.2 | | | 35.4 |
| **t_1/2z_ (h)** |  |  | | |  |
| Mean (SD) | 3.0 (2.9) | 5.6 (2.8) | | | 4.6 (0.9) |
| %CV | – | – | | | – |

AUC_∞_, area under the curve from time zero to infinity; AUC_τ,ss_, area under the curve during a dosing interval, at steady state; AUC_24_, area under the curve over 24 hours; AUC_last_, area under the curve from time zero to time of last measurable concentration; C_max_, maximum plasma drug concentration; C_max_,_ss_ maximum plasma drug concentration during a dosing interval, at steady state; CV, coefficient of variation; Geo, geometric; SD, standard deviation; t_1/2z_, elimination half-life; T_max_, time to maximum concentration; T_max,ss_, time to maximum concentration at steady state.
