# Supplementary material for: Evaluating the Safety, Tolerability, and Disposition of Trazpiroben, a D2/D3 Receptor Antagonist: Phase I Single‐ and Multiple‐Ascending Dose Studies in Healthy Japanese Participants
Source: Clin Pharmacol Drug Dev. 2021 Dec 29;11(6):695–706. doi: 10.1002/cpdd.1057 (PMC9303893; doi:10.1002/cpdd.1057)
Supplement: Supplementary file 8 — Supporting information [file CPDD-11-695-s008.docx]

# Supplementary Table 3. Urine PK Parameters of Trazpiroben (a) and M23 (b) in Japanese Participants

(a)

| **Visit**  Variable | **Trazpiroben dose** | **N** | **Mean (SD)** |
| --- | --- | --- | --- |
| **Day 1** |  |  |  |
| Ae_24_ (μg) | 10 mg | 6 | 160.0 (29.8) |
|  | 50 mg | 6 | 741.2 (114.1) |
|  | 100 mg | 6 | 1571 (621.6) |
| f_e,24_ (%) | 10 mg | 6 | 2.0 (0.4) |
|  | 50 mg | 6 | 1.8 (0.3) |
|  | 100 mg | 6 | 1.9 (0.8) |
| CL_R_ (L/h) | 10 mg | 6 | 11.8 (2.5) |
|  | 50 mg | 6 | 10.3 (1.9) |
|  | 100 mg | 6 | 9.7 (2.6) |
| **Day 7** |  |  |  |
| Ae_24_ (μg) | 10 mg | 6 | 198.7 (28.1) |
|  | 50 mg | 6 | 895.8 (179.7) |
|  | 100 mg | 6 | 2155 (358.7) |
| f_e,24_ (%) | 10 mg | 6 | 2.4 (0.3) |
|  | 50 mg | 6 | 2.2 (0.4) |
|  | 100 mg | 6 | 2.6 (0.4) |
| CL_R_ (L/h) | 10 mg | 6 | 12.2 (1.8) |
|  | 50 mg | 6 | 10.0 (1.9) |
|  | 100 mg | 6 | 11.5 (1.5) |

b)

| **Visit**  Variable | **Trazpiroben dose** | **N** | **Mean (SD)** |
| --- | --- | --- | --- |
| **Day 1** |  |  |  |
| Ae_24_ (μg) | 10 mg | 6 | 13.3 (6.5) |
|  | 50 mg | 6 | 64.6 (23.7) |
|  | 100 mg | 6 | 135.2 (80.9) |
| f_e,24_ (%) | 10 mg | 6 | 0.2 (0.1) |
|  | 50 mg | 6 | 0.2 (0.1) |
|  | 100 mg | 6 | 0.2 (0.1) |
| CL_R_ (L/h) | 10 mg | 6 | 9.2 (2.8) |
|  | 50 mg | 6 | 8.0 (1.6) |
|  | 100 mg | 6 | 7.8 (2.0) |
| **Day 7** |  |  |  |
| Ae_24_ (μg) | 10 mg | 6 | 16.1 (7.3) |
|  | 50 mg | 6 | 78.8 (29.4) |
|  | 100 mg | 6 | 237.7 (109.0) |
| f_e,24_ (%) | 10 mg | 6 | 0.2 (0.1) |
|  | 50 mg | 6 | 0.2 (0.1) |
|  | 100 mg | 6 | 0.3 (0.1) |
| CL_R_ (L/h) | 10 mg | 6 | 9.1 (2.2) |
|  | 50 mg | 6 | 7.7 (1.6) |
|  | 100 mg | 6 | 8.7 (1.0) |

Ae_24_, cumulative amount of unchanged drug excreted into the urine over 24 hours; CL_R_, renal clearance of the drug from plasma; f_e,24_, fraction of administered drug excreted in urine over 24 hours; N: total patient number; Q1, quartile 1; Q3, quartile 3; SD, standard deviation.
